# Acupuncture Therapy as an Evidence-Based Nonpharmacologic Strategy for Comprehensive Acute Pain Care: The Academic Consortium Pain Task Force White Paper Update

**DOI:** 10.1093/pm/pnac056

**Published:** 2022-06-17

**Authors:** Arya Nielsen, Jeffery A Dusek, Lisa Taylor-Swanson, Heather Tick

**Affiliations:** Department of Family Medicine & Community Health, Icahn School of Medicine at Mount Sinai, New York, New York; University Hospitals, Connor Whole Health, Cleveland Medical Center, Cleveland, Ohio; Department of Family Medicine and Community Health, Case Western Reserve University, Cleveland, Ohio; University of Utah College of Nursing, Salt Lake City, Utah; Department of Family Medicine, and Anesthesiology & Pain Medicine, University of Washington School of Medicine, Seattle, Washington, USA

## Abstract

**Background:**

A crisis in pain management persists, as does the epidemic of opioid overdose deaths, addiction, and diversion. Pain medicine is meeting these challenges by returning to its origins: the Bonica model of multidisciplinary pain care. The 2018 Academic Consortium White Paper detailed the historical context and magnitude of the pain crisis and the evidence base for nonpharmacologic strategies. More than 50% of chronic opioid use begins in the acute pain care setting. Acupuncture may be able to reduce this risk.

**Objective:**

This article updates the evidence base for acupuncture therapy for acute pain with a review of systematic reviews and meta-analyses on postsurgical/perioperative pain with opioid sparing and acute nonsurgical/trauma pain, including acute pain in the emergency department.

**Methods:**

To update reviews cited in the 2018 White Paper, electronic searches were conducted in PubMed, MEDLINE, CINAHL, and the Cochrane Central Register of Controlled Trials for “acupuncture” and “acupuncture therapy” and “acute pain,” “surgery,” “peri-operative,” “trauma,” “emergency department,” “urgent care,” “review(s) ,” “systematic review,” “meta-analysis,” with additional manual review of titles, links, and reference lists.

**Results:**

There are 22 systematic reviews, 17 with meta-analyses of acupuncture in acute pain settings, and a review for acute pain in the intensive care unit. There are additional studies of acupuncture in acute pain settings.

**Conclusion:**

The majority of reviews found acupuncture therapy to be an efficacious strategy for acute pain, with potential to avoid or reduce opioid reliance. Future multicenter trials are needed to clarify the dosage and generalizability of acupuncture for acute pain in the emergency department. With an extremely low risk profile, acupuncture therapy is an important strategy in comprehensive acute pain care.

## Introduction

Medical pain management is in crisis. Pain is pervasive and has been inadequately addressed by strategies including the escalation of prescription opioids that created a tragic increase in overdose deaths, addiction, and diversion. The rising costs of pain care and managing adverse effects of that care have prompted action from state and federal agencies including the Veterans Health Administration (VHA) [1], the U.S. Department of Veterans Affairs (VA) and the Department of Defense (DOD) [2], the National Institutes of Health (NIH) [3], the U.S. Food and Drug Administration (FDA) [4], and the Centers for Disease Control and Prevention (CDC) [5]. There has been pressure for pain medicine to shift away from an overreliance on opioids and overutilized procedures and surgeries toward more comprehensive pain care that includes evidence-based nonpharmacologic strategies. Comprehensive pain care programs build on the foundation developed by John Bonica, the father of pain medicine, and are further supported by the intervening decades of research into nonpharmacologic strategies [6].

The evidence base for acupuncture therapy is extensive. Acupuncture is supported or recommended as part of comprehensive pain care by the U.S. Agency for Healthcare Research and Quality (AHRQ) [7], the U.S. Food and Drug Administration (FDA) [8], the U.S. Department of Health and Human Services (HHS) [9], and the Joint Commission TJC [10, 11]. The American Academy of Family Physicians endorsed the American College of Physicians (ACP) Guidelines recommending acupuncture as a first option for acute, subacute, and chronic low back pain (LBP) [12, 13]. A retrospective claims-based study found that initial visits to chiropractors, physical therapists, or acupuncturists for new-onset LBP substantially decreased early and long-term use of opioids [14]. A retrospective analysis of 427,966 patients with new-onset neck pain and back pain found that patients who saw these conservative therapists, compared with those who had primary care visits, were 72–91% less likely to fill an opioid prescription in the first 30 days and 41–87% less likely to continue filling prescriptions for 1 year [15]. Although the claims data do not indicate pain severity or duration, the authors controlled for comorbidities through the use of the Elixhauser Index, which has served as a proxy for complexity in other studies. Conversely, people with acute neck pain who initially saw an emergency department (ED) physician had the highest odds of opioid use during the first 30 days [15].

Perioperative opioid prescribing has been associated with persistent opioid use after surgery [16]. As of 2017, more than 50% of chronic opioid use began in the acute care setting, after surgery, or for treatment of acute injury-related pain [17]. The probability of long-term opioid use increases after as few as 5 days of prescribed opioids as the initial treatment of pain; the probability that patients will remain on opioids for the long term is the highest when treatment is initiated with long-acting opioids [18]. Although opioid overdose deaths began to decrease slightly in 2018 [19], they rose to an all-time high in 2019 [20]. By the end of 2020, accelerated by the coronavirus disease 2019 (COVID-19) pandemic, opioid overdose deaths increased by more than 35% from 2019 [20–22].

Tapering, which may or may not include cessation of opioids, has been a key strategy to reduce the risks and harms of opioids. However, dose tapering is often a lengthy and time-consuming process, and attempts at rapid tapering of doses are associated with unintended consequences, risks, and patient suffering [23, 24]. Even low-dose opioid use for more than 3 months increases the risk of addiction 15-fold [25]. Sustained opioid dependence is also associated with declining function and exacerbation of pain states (hyperalgesia) secondary to mechanisms of the action of opioids [26, 27]. Reducing reliance on opioids for acute pain in the ED depends not only on avoidance of prescribing opioids [25, 28] but also on supporting access to effective acute pain care strategies. Transforming the system of pain care to a responsive comprehensive model necessitates that options for treatment and collaborative care be evidence based and include effective nonpharmacologic strategies that have the advantage of reduced risks of adverse events and addiction liability. The 2018 Academic Consortium White Paper [6] detailed the historical context and magnitude of the current pain problem, including individual, social, and economic impacts, as well as the challenges of pain care for patients and a health care workforce engaging in prevalent strategies that are not entirely based on current evidence [29].

The present article is the first in a series of articles to update the evidence base for nonpharmacologic therapies that are effective in acute pain [6]. The present article provides a review of reviews and meta-analyses of acupuncture for acute pain, encompassing postsurgical/perioperative pain with opioid sparing and acute nonsurgical/trauma pain, including acute pain in the ED and primary care setting.

## Methods

To update and detail the literature cited in the 2018 White Paper [6] on nonpharmacologic strategies for comprehensive pain care, specifically research on acupuncture for acute pain, a search for systematic reviews (SRs), either with meta-analyses (SRMs) or without meta-analyses, was conducted for articles published through December 2020 in PubMed, MEDLINE, CINAHL, and the Cochrane Central Register of Controlled Trials for “acupuncture” and “acupuncture therapy” and “acute pain,” “surgery,” “peri-operative,” “trauma,” “emergency department,” “urgent care,” “review(s) ,” “systematic review,” “meta-analysis,” with additional manual review of titles, links, and reference lists. We included articles published in peer-reviewed journals for which access to data was possible, and we excluded reviews when data or original articles could not be accessed (e.g., articles published in Chinese). Additionally, we detail research articles published since the publication of the SRs that would help inform researchers, clinicians, and policy makers on the current state of research.

## Results

Twenty-two SRs were selected for review, including 17 with meta-analyses of acupuncture in acute pain settings and a review for pain in the intensive care unit (ICU). Details of SRs and SRMs are represented in Tables 1, 2, 3, 6, and 7. Details of additional studies of acupuncture in acute pain settings are included in Tables 4 and 5.

**Table 1:** Acupuncture for acute postoperative pain: SRs with and without meta-analyses

Authors	Modality	SR	Meta-Analysis	Setting, Condition, Number	Comparators	Results	Reduced Analgesics, Including Opioids	Quality and Recommendation
Sun et al. 2008 [36]	RCTs: 6 e-stim 4 manual acupuncture 3 ear acupuncture 1 *Capsicum* plaster 1 acupressure	15 trials	10 trials	Surgery type: Abdominal (6) Maxillo-facial (2) Knee (2) Hemorrhoid (1) Back (1) Thoracotomy (1) Hip arthroplasty (1) Molar tooth extraction (1) n = 1,166	Sham and usual care: 10 general anesthesia 4 local anesthesia 1 unreported	Pain intensity at 8, 24, and 72 hours: 8 hours: WMD −14.57 mm (95% CI −23.02 to −6.13). 24 hours: WMD −5.59 mm (95% CI −11.97 to 0.78). 72 hours: WMD −9.75 mm (95% CI −13.82 to −5.68). May not be clinically relevant. Opioid side effects as NNT: nausea NNT = 6; vomiting no difference; pruritus NNT = 13; dizziness NNT = 6; sedation NNT = 11; urinary retention NNT = 5. Opioid sparing is clinically meaningful. No significant AEs.	21% decrease at 8 hours, 23% at 24 hours, and 29% at 72 hours. Opioid-sparing effect considered clinically relevant.	Overall SOE not assessed. Moderate reduction in pain intensity that may or may not be clinically relevant. Relative reduction in opioid consumption of 21–29%, which is considered clinically relevant. Recommend acupuncture as adjuvant, and further research is needed.
Asher et al. 2010 [38]	Ear acupuncture	17 RCTs: 8 perioperative; 4 acute pain; 5 chronic pain	8 trials; 5 perioperative acute pain	17 trials n = 1,009; perioperative n = 551	Sham and usual care	Pain reduction: SMD was 1.56 (95% CI 0.85 to 2.26), indicating that on average, the mean decrease in pain score for the auriculotherapy group was 1.56 standard deviations greater than the mean decrease for the control group.	Analgesic consumption was lower in tx group: SMD 0.54 (95% CI 0.30 to 0.77); 5 studies.	Overall SOE: moderate. Recommend auriculotherapy as reasonable adjunct for pain, especially postoperative pain and for patients with intolerance to pain medications.
Liu et al. 2015 [34]	APS= body acupuncture, e-stim, acupressure, ear seeds, *Capsicum* plaster therapy	59 trials	39 trials: pooled trial subgroups n = 2,097 acupuncture	Surgery: abdominal, knee, oral, cardiac, hemorrhoid, C-section; n = 4,402	Sham/placebo control (36 trials) and usual care (n = 2,305): standard anesthetic and postoperative analgesia regimens used in all trials.	Improved VAS scores, especially for abdominal, cardiac, and C-section surgery.	APS reduced analgesic requirement in postoperative patients without AEs.	Overall SOE: Level I evidence for body point acupuncture stimulation reducing postoperative pain intensity and patient’s analgesic need. Overall SOE: Level II for abdominal surgery, Level III for cardiac and C-section. APS favorable, low risk, low complication rate, economical. Ongoing research needed.
	Ear point stimulation	14 trials	12 trials	Postsurgical (n not stated)	Sham/placebo and usual care	Reduced postoperative pain intensity.	Reduced analgesic requirement without AEs.	Overall SOE: Level I evidence for ear point stimulation reducing postoperative pain intensity.
	Ear and body acupuncture	7 trials	7 trials	Postsurgical (n not stated)	Sham/placebo and usual care	Reduced postoperative pain intensity.	Reduced analgesic requirement without AEs.	Overall SOE: Level II evidence for reduction of postoperative pain for mixed body and ear acupuncture.
Cho et al. 2015 [48]	E-stim at nonpenetrating acupuncture point; ear acupressure; manual acupuncture	2 acupoint e-stim 1 ear acupressure 2 manual acupuncture	5 trials	Postoperative back surgery, n = 410	vs sham (3) vs usual care (2)	Acupuncture reduced acute postoperative pain in first 24 hours.	Reduced opiate demand similar to sham at 24 hours. Reduced opiate dose when compared with usual care.	Overall SOE: moderate. Encouraging, but larger pragmatic trials are needed.
Chou et al. 2016 [41]	(Acupuncture as one reviewed modality)	2 superficial intradermal needles thoracic surgery (abbreviated tx) 1 classical acupuncture lumbar disc surgery 1 knee surgery acupuncture vs proximal needling 1 knee surgery post-anesthesia 1 postoperative (1994) active placebo	6 trials	Preoperative, intraoperative, postoperative (n not stated)	Active comparators not inert controls, potentially leading to underestimation of the value of acupuncture.	Inclusion of only 6 trials, 2 with superficially retained needles considered an abbreviated tx. Trials dated from 1994 to 2008.	Not reported.	Overall SOE: “insufficient evidence.” Considered safe. Do not encourage or discourage acupuncture for surgical pain.
Fuentealba et al. 2016 [49] (Chile)	Acupuncture and ear acupuncture	5 trials 2 SRs	No meta-analysis	Postoperative tonsillectomy, knee replacement, dental surgery (n not stated)		Reduced pain by 36% (at 20 minutes) and 22% (at 2 hours) for tonsillectomy. Reduced pain by 2% for TKA. Reduced pain by 24% (at 2 hours) for dental procedures.	42% reduced analgesic consumption (at 2 hours).	Overall SOE: not assessed. No meta-analysis because of study heterogeneity. Acupuncture may be useful to manage postoperative pain. More study needed.
Wu et al. 2016 [35]	Acupuncture EA TEAS Acupuncture point e-stim	13 RCTs: 4 acupuncture, 4 EA, 5 TEAS	11 RCTs: 2 acupuncture, 4 EA, 5 TEAS	Postoperative, n = 682	“Control” arms not detailed	Conventional acupuncture and TEAS lowered postoperative pain on first postoperative day.	TEAS reduced opioid use.	Overall SOE: moderate. Findings support use of acupuncture as adjuvant therapy for postoperative pain.
Tedesco et al. 2017 [37]	Acupuncture	4 of 77 RCTs on acupuncture	3 of 39 RCTs on acupuncture	Post-TKA, n = 230 of 2,391	Sham or nothing as comparator	Significant improvement for acupuncture vs control group with MD−1.14 (95% CI −1.90 to −0.38), P= 0.003 on VAS at 6 months.	Modest but clinically significant evidence that acupuncture is associated with reduced and delayed opioid consumption.	Overall SOE: low for pain relief. Acupuncture studies: less risk of bias. Findings support use of acupuncture after TKA.
Murakami et al. 2017 [40]	Ear acupuncture and electro ear acupuncture.	10 trials	3 trials pain intensity as primary measure, n = 349; 6 trials evaluating analgesic requirement, n = 303	Acute care and postoperative; n = 700	4 analgesics, 5 sham acupuncture, 1 distraction	Ear acupuncture was superior to comparator (MD −0.96 [95% CI −1.82 to −0.11]), but the MD was small.	Reduced analgesic need (fentanyl, piritramide, desflurane, papaveretum, ibuprofen); acupuncture was superior (MD −1.08 [95% CI −1.78 to −0.38]), with a small MD.	Overall SOE: low to moderate. Immediate pain relief equivalent to analgesics and to 48 hours. Promising modality for pain reduction in 48 hours with low side effect profile.
Ye et al. 2019 [39]	Perioperative auricular therapies (includes auricular acupuncture, auricular point buried bean, auricular massage, auricular magnetic therapy, and auricular moxibustion)	9 trials	7/9	THA; n = 605	Measures: VAS, intraoperative amount fentanyl, time to first analgesic request, nausea and vomiting, perioperative bradycardia, perioperative hypotension. 2/9 tracked NSAIDs; sham acupuncture 4/9.	Perioperative VAS value of the intervention group was significantly lower than that of control group at different time points in patients after THA (6 hours to 7 days). Observation time points: Postoperative 12 hours: SMD −1.03 (95% CI −1.51 to −0.55), *P* < 0.001. Postoperative 24 hours: SMD −0.95 (95% CI −1.53 to −0.37), *P* = 0.001, *P* = 0.08. Postoperative 48 hours: SMD −0.89 (95% CI −1.48 to −0.30), *P* = 0.003. Postoperative 72 hours: SMD −0.79 (95% CI −0.92 to −0.66), *P* < 0.001. Postoperative 5 days: SMD −0.60 (95% CI −0.94 to 0.26), *P* < 0.001. Postoperative 7 days: SMD −0.68 (95% CI −1.01 to −0.35), *P* < 0.001.	Acupuncture group had lower values than the control group (SMD −0.73 [95% CI −1.09 to −0.36], *P* = 0.0001). Evidence of auricular therapies on postoperative pain and intraoperative body mass–adjusted fentanyl amount for the patients after THA was affirmative but did not show prolonged time to first analgesic request or the incidence of postoperative medication-related complications.	Overall SOE: low but affirmative for auricular therapies and post-THA pain. Verification is needed in future multicenter trials.
Zhu et al. 2019 [44]	17 trials: Distal: 9 EA, 1 TEAS, 1 manual acupuncture, 3 acupressure, 1 auricular, 1 *Capsicum* plaster 17 trials peri-incision: TENS using surface electrodes. 1 trial distal and local	35 trials (30 in English, 5 in Chinese)	15/17 distal 11/17 peri-incision	Inpatient. Distal: n = 959 Peri-incisional: n = 805	Distal trials: 5 sham 7 nonactive tx 5 both Peri-incision TENS trials: 11 sham 3 nonactive tx 3 both	Pain intensity at rest at 4, 12, 24, and 48 hours: 4 hours: MD −11.82 mm (95% CI: −15.47 to −8.16), *I*^2^ 64%. 12 hours: MD −11.92 mm (95%CI: −13.58 to −10.26), *I*^2^ 84%. 24 hours: MD −7.14 mm (95% CI −8.95 to −5.13), *I*^2^ 40%. 48 hours: MD −9.45 mm (95% CI −12.41 to −6.50), *I*^2^ 68%. Peri-incisional stimulation also showed beneficial effects compared with controls: 4 hours: MD −10.70 mm (95% CI −15.32 to −6.0), *I*^2^ 45%. 12 hours: MD −13.52 mm (95% CI −15.25 to −11.78), *I*^2^ 92%. 24 hours: MD −7.13 mm (95% CI −12.38 to −1.88), *I*^2^ 65%. 48 hours: −10.32 mm (95% CI −14.28 to −6.37), *I*^2^ 47%. Distal acupuncture showed better effects than controls at: 4 hours: MD −26.49 mm (95% CI −35.56 to −17.42), *I*^2^ 83%. 24 hours: distal: −17.48 mm (95%CI: −23.25 to −11.70), *I*^2^ 88%. 48 hours: distal: −16.61 mm (95% CI −21.95 to −11.62), *I*^2^ 82%. Peri-incisional stimulation also showed beneficial effects compared with their controls at: 4 hours: MD −4.46 mm (95% CI −13.62 to 4.70), *I*^2^ 0%. 24 hours: −9.53 mm (95% CI −14.19 to −4.87), *I*^2^ 0%. 48 hours: −14.02 mm (95% CI −19.06 to −8.98), *I*^2^ 0%. Subgroup analysis showed no difference between peri-incisional or distal stimulation on postoperative pain reduction. Both reduced pain at rest compared with their controls. Distal had better effect for pain on movement or cough.	Both reduced postoperative opioid consumption at 24 hours compared with sham. Peri-incisional stimulation was superior in reducing opioid consumption at 24 hours, whereas distal acupoint stimulation reduced opioid-related adverse effects, including nausea and dizziness. The pain intensity on movement at postoperative 4 hours was lower in distal stimulation. Both reduced postoperative opioid consumption at 24 hours.	Overall SOE: moderate. Perioperative distal acupoint or peri-incisional stimulation is safe and effective for postoperative pain and opioid sparing. They could be alternative or adjunct analgesic intervention. More studies, larger sample size, and direct comparison needed in future.

AE = adverse event; APS = acupuncture point stimulation; CI = confidence interval; EA = electroacupuncture; ear acupuncture = auricular acupuncture; e-stim = electrical stimulation; MD = mean difference; NNT = number needed to treat; NSAIDs = non-steroidal anti-inflammatory drugs; SMD = standard mean difference; SOE = strength of evidence; TEAS = transcutaneous acupoint electric stimulation; TENS = transcutaneous electrical nerve stimulation; THA = total hip arthroplasty; TKA = total knee arthroplasty; tx = treatment; VAS = visual analog scale; WMD = weighted mean difference.

**Table 2:** Acupuncture for acute traumatic and ED acute pain: SRs with and without meta-analyses

Authors, Year	Modality	SR	Meta-Analysis	Setting, Condition, Number	Outcomes/Comparators	Results	Quality and Recommendation
Kim et al. 2013 [50]	Needle insertion including auricular points	SR 2 RCTs 2 OBS	NA	Acute pain syndromes and nonpenetrating injuries of the extremities (cardiac, including heart attack) ED setting n = 225	Pain VAS or NRS Physiological parameters (respiratory rate, heart rate, systolic and diastolic blood pressure) Medication consumption Length of stay in ED Patient satisfaction with the tx Time points: immediate post-tx acupuncture plus UC vs UC alone Safety, effectiveness, and feasibility of acupuncture in the ED	Studies showed it feasible to provide acupuncture in the ED and suggested further study to test the role of acupuncture in the ED.	Overall SOE not assessed. Internal validity assessed with Cochrane Risk of Bias Tool but no rating provided. Current evidence found in study was insufficient to accept or refute the use of acupuncture in the ED. Future studies should address the process and cost-related benefits of acupuncture use in the ED: future research with large RCTs to evaluate effectiveness of acupuncture in the ED and future OBS on the safety and acceptability of acupuncture to ED staff and patients. Current evidence is insufficient to provide any recommendation of acupuncture in the ED setting.
Jan et al. 2017 [54]	Auricular therapies, including auricular acupuncture and auricular pressure	SRM 6 RCTs	Meta-analysis 4 RCTs, n = 286 auricular therapies vs sham, n = 127 Auricular therapies+SAC vs SAC alone n = 154 auricular therapies alone or +SC vs control as sham alone or +SC, n = 271	Acute pain management n = 458	Pain (PS-10) difference in: auricular acupuncture vs sham; AA as an adjunct to other analgesia (AdjA) vs SAC; auricular acupuncture vs SAC Medication usage Patient satisfaction	auricular acupuncture vs sham: SMD of 1.69 (CI 95%: 0.37–3.01) WMD 2.47 (CI 95% 1,79–3.16) AdjA vs SAC: SMD 1.68 (CI 95% 1.18–2.18) WMD 2.84 (CI: 95% 1.45–4.22) 1 RCT showed reduction in NSAID usage for sore throats with reduced mean number of doses at 6 hours, 24 hours, and 48 hours. In OBS AdjA, 62% of respondents said that they “would have the same treatment again,” and 71% reported they were either mostly satisfied or very satisfied.	Overall SOE not assessed. Ear acupuncture has limited evidence of effectiveness for acute pain in the ED setting as standalone tx as an adjunct. Future studies needed with comparator group of acupuncture vs SAC. Future studies needed with patient satisfaction as secondary outcome. Future studies needed assessing various techniques of ear acupuncture, ear vs body acupuncture, and utilization of certified acupuncturists vs non-acupuncturists. Further testing using acupuncture vs SAC with medication usage as secondary outcome.
Jan et al. 2017 [51]	Acupuncture (26), auricular therapy (3), EAS (1)	SRM 19 RCTs and 11 OBS in SR	14 RCTs n = 1,210	Acute pain management in the ED setting n = 3,169 SR n = 1,210 meta-analysis 11 OBS Migraine, hip fractures, biliary colic, acute LBP, sore throat. 4 spinal pain, 3 mixed pain, 3 limb fractures, 3 migraine, 3 renal colic, 11 traditional acupuncture, 5 ear acupuncture (4 BFA)	Pain (PS-10) difference: acupuncture vs sham, acupuncture vs SAC, acupuncture as adjunct to other analgesia (AdjA) vs SAC Pain scored recorded within 240 minutes of tx Analgesia use Patient satisfaction Time and cost of acupuncture	Acupuncture vs sham: SMD 1.08 (95% CI = 0.62–1.54), WMD of 1.60 (95% CI = 0.98–2.23). (both favoring acupuncture) acupuncture vs SAC: (acupuncture comparable to SAC) AdjA vs SAC alone: SMD 1.68 (95% CI 1.18–2.18), WMD 2.84 (95% CI 1.45–4.22) AdjA more effective than SAC (without sham). Patient satisfaction, reported in 5 RCTs, showed improvement compared with sham on 100-point scale. 5 OBS measured patient satisfaction; all reported improvement with AC. 3 RCTs quoted costs of acupuncture consumables less than $5 per patient; 3 other RCTs stated acupuncture is “low-cost treatment.” Acupuncture provided statistically significant, clinically meaningful, and improved levels of patient satisfaction with respect to pain relief in the emergency setting.	Overall SOE not assessed. Acupuncture appears to provide effective analgesia for some acute pain conditions in the ED, while being noninferior to selected analgesia medications. Low-cost, low-risk, and patient-satisfying therapy. Effectiveness in reducing analgesic medication use is uncertain. Future RCTs might measure the NNT for 30–50% pain reduction or “adequate analgesia,” which has better correlation to patient satisfaction. More RCTs where AdjA is compared with SAC. More investigation into other pain conditions with acupuncture vs SAC, ear vs body acupuncture, and acupuncture delivered by ED health providers vs qualified acupuncturists.
Chia et al. 2018 [52]	Acupuncture, auricular acupuncture, EAS	SR 6 RCTs	NA	Acute clinical conditions in the ED, including acute pain, HTN, and cardiac arrest n = 651	Pain= most frequently assessed outcome Effective/success rate of tx based on individual study criteria	Acupuncture vs sham for acute pharyngitis: Acupuncture 44.4% vs sham 10.5%, at relieving pain. Acupuncture vs standard ED care for acute pain: Acupuncture was more effective and faster pain control compared with intravenous morphine, success rate acupuncture 92% vs 78%. EAS vs standard ED care for acute pain significant reduction in mean VAS score seen in both groups (acupuncture group 25.90 ± 17.62; conventional ED care group 22.18 ± 24.09). Acupuncture as an adjunct to standard ED care for acute pain syndrome auricular acupuncture +SC better than SC alone in immediate pain control, with 2.18 mean difference in NRS pain reduction.	Overall SOE not assessed. Further studies evaluating clinical efficacy and effectiveness of acupuncture in the ED are needed. Multicenter RCTs are needed.
Sakamoto et al. 2018 [53]	Acupuncture, auricular, scalp acupuncture	SRM 10 acupuncture studies 5 RCTs 1 cohort 4 case series	4/9 direct modality acupuncture RCTs: n = 525	Acute pain in the ED n = 724	Pain= most frequently assessed outcome with VAS or NRS: acupuncture vs no intervention, acupuncture no comparator, acupuncture vs sham, acupuncture vs titrated morphine, acupuncture vs intravenous acetaminophen vs intramuscular diclofenac	Acupuncture decreased pain immediately until ED discharge (4 RCTs, 1 cohort, 4 case series) and improved nausea, anxiety, time to pain resolution, and adverse effects. Pain decrease similar to control immediately, 30 minutes, and 24 hours after acupuncture (3 RCTs). 84% of patients reported benefit, 52–82% would use again, nearly all patients reported high satisfaction, with >50% reporting highest satisfaction.	Overall SOE not assessed. Studies addressing feasibility of implementation, opioid usage, and efficacy in terms of multidimensional functional outcomes are warranted. Interventions have potential to improve acute pain management and patient satisfaction and improve patient outcomes and quality of life, while reducing overall ED utilization and length of stay.
Liu et al. 2020 [64]	Acupuncture; acupuncture+ Chinese herbs/tincture; acupuncture+ massage; acupuncture+ RICE; acupuncture + medications	SRM 17 trials Acute ankle sprain	Acupuncture+ RICE vs RICE n = 143 Acupuncture+ dimethyl sulfoxide vs dimethyl sulfoxide alone n = 87 Acupuncture+ Chinese medicine vs Chinese medicine alone n = 530	Outpatient care Tx 1–21 days; acute ankle sprain n = 1528	Kofoed Ankle Score. VAS, duration of pain, use of analgesics, ankle circumference “Effective rate,” “cure rate” (Chinese studies) Adverse events. Comparators: no tx, placebo, or traditional therapies for acute ankle sprain involve nonsteroidal anti-inflammatory drugs, RICE (rest, ice, compression, and elevation), functional support, exercise, manual mobilization, etc.	Meta-analysis favored acupuncture vs no tx, vs massage, vs ice/hot pack+ Chinese medicine, vs infrared radiation, and vs RICE but not vs dimethyl sulfoxide. Acupuncture plus dimethyl sulfoxide vs dimethyl sulfoxide alone, acupuncture plus massage vs massage alone, acupuncture plus RICE vs RICE alone.	Overall SOE not assessed. Acupuncture could be beneficial for acute ankle sprain; more large-scale well-designed RCTs warranted.

AA = auricular acupuncture; AP = auricular pressure (as in ear seeds); AdjA =  adjunct acupuncture; EAS = electroacupuncture stimulation (e-stim on needles inserted at acupoints); HTN = hypertension; NA = not applicable; NNT= number needed to treat; NRS = numerical rating scale; OBS = observational study; PS-10 = difference in standardised pain scores out of 10; RICE = rest, ice, compression, elevation; SAC = standard analgesic care; SC = standard care; SOE = strength of evidence; tx= treatment; UC = usual care; VAS = visual analog scale.

**Table 3.** Nonpharmacologic therapy including acupuncture for acute pain in the ICU

Authors, Year	Modality/ Kind of Study, Number	Setting and Types of Pain	Outcomes/Comparators	Results	Recommendation
Sandvik et al. 2020 [55]	Review of 12 studies Hypnosis, massage, distraction, relaxation, spiritual care, harp music, music therapy, listening to natural sounds, passive exercise, acupuncture (n = 576), ice packs, and emotional support.	ICU Pain provoking tissue damage, disease, surgery/medical and nursing procedures	Measures: VAS (7); NRS (2); Edmonton Symptom Assessment (1); observational pain scale and BPS (2). Various designs: quasi-experiments with control groups (6); a tx with matched controls (1); case-controlled study with pre- and post-tests (1); an intervention without control using pre- and post-tests (1); qualitative descriptive (2); crossover design with randomization (1)	Reduced pain intensity from hypnosis, acupuncture, and natural sounds	Overall SOE not assessed. Suggest use of comprehensive multimodal interventions to investigate effects of nonpharmacologic tx protocols on pain, intensity, pain proportion, and impact on opioid consumption and sedation requirements.

BPS = Brief Pain Scale; NRS = numerical rating scale; SOE = strength of evidence; tx= treatment; VAS = visual analog scale.

**Table 4:** Acupuncture RCTs for acute pain: inpatient, surgery, ICU, and ED

Authors/year	Modality/Kind of Study/n	Setting and Types of Pain	Intervention and Comparators	Results
Zheng et al. 2012 [65]	Acupuncture or EAS Exploratory study n = 45	ICU Pain of intubated patients (under mechanical ventilation)	UC n = 15 UC plus acupuncture n = 15 (v24, Yin tang) de qi and 6 hours UC plus EAS n = 15 (GV 24, Yintang); 30 minutes on and off / 6 hours	EAS markedly reduced dosage of sedative drug (midazolam) needed for pain/discomfort of mechanical intubation.
Murugesan et al. 2017 [66]	Acupuncture Double-blind RCT n = 157	Outpatient, acute dental pain, irreversible pulpitis, tooth extraction	Acupuncture needles 15–20 minutes Classical acupuncture+ placebo tablet (n = 53) Sham acupuncture+ placebo tablet (n = 52) Sham acupuncture+ ibuprofen (n = 52) VAS before and after tx: 15, 20. 45, and 60 minutes Follow-up: 12, 24, and 48 hours	Acupuncture+ placebo tablet showed statistically significant lower pain values, no difference between either sham arm including with ibuprofen. acupuncture+ placebo tablet higher % no pain on follow-up= statistically significant to comparison groups.
Cohen et al. 2017 [56]	Acupuncture Equivalence, noninferiority RCT n = 528	Multicenter ED Acute LBP n = 270 Migraine n = 92 Ankle sprain n = 166	Prescribed acupuncture protocol per clinical condition Acupuncture alone (n = 177) Acupuncture+ pharm (n = 178) Pharm alone (n = 173) Pharm: Diazepam 5 mg, Hartmann’s solution, paracetamol 1 g, paracetamol 500 mg+ codeine 30 mg, tramadol 40–100 mg, dextropropoxyphene 32.5 mg+ paracetamol 325 g, ibuprofen 400 mg, diclofenac 50 mg, indomethacin 100 mg as needed. After 1 hour, second line: morphine 2.5-mg intravenous boluses, chlorpromazine 25 mg in 1,000 mL normal saline. VNRS Scale T0 and at every hour until discharge; functionality by Oswestry Low Back Disability Questionnaire, 24-Hour Migraine Quality of Life Questionnaire, or Patients Global Assessment of Ankle Injury Scale at T48 Acceptability T1, T48; health resource use, length of stay, readmission rate, additional analgesia.	Acupuncture analgesia comparable to pharm for acute back pain and ankle sprain. Three arms similarly effective at reducing pain at T1 but less than 40% of participants had reduction of pain of 2 points or more at T1 where more than 80% had pain of 4 or more. By T48, 61% of acupuncture alone, 57% combined, and 52% of pharm alone were definitely willing to repeat tx. Mild AE in each arm. Safe and acceptable.
AminiSaman et al. 2018 [67]	Double-blind RCT n = 60	OR: spinal anesthesia for trans-urethral lithotripsy surgery	TENS (n = 30) electrodes applied to GV channel at point between lumbar 3–4 and lumbar 5–S1 (extra point: M-BW-25: Shiqizhuixia) vs control of no intervention (n = 30)	Intervention reduced pain of spinal anesthesia; duration of spinal anesthesia implementation procedure by physician in the intervention group was significantly shorter than that of the control group.
AminiSaman et al. 2018 [68]	TENS at acupuncture points RCT n = 50	ICU Pain of intubated patients (under mechanical ventilation)	Li 4 and St 36 bi 30 min, 4×/24 hours vs sham (same device, not activated)	Reduction in pain and analgesic and sedation medication.
Fox et al. 2018 [69]	Ear acupuncture n = 30	ED Acute LBP	Ear acupuncture (n = 15) Standard care (n = 15)	Acupuncture was feasible and effective in reducing pain intensity; comparable outcomes in “get up and go test”
Beltaief et al. 2018 [58]	Acupuncture n = 115	ED Acute renal colic	Acupuncture (n = 54) vs titrated morphine (n = 61)	Time to 50% pain reduction: acupuncture (14 minutes) vs morphine (28 minutes). Acupuncture associated with much faster and deeper analgesic effect. Acupuncture had better tolerance profile than titrated morphine.
Crawford et al. 2019 [70]	Ear acupuncture BFA n = 233	Lower-extremity surgery acute pain	Modified BFA (n = 81) (right ear including cingulate gyrus, thalamus, omega 2, shen-men, and point zero) Sham acupuncture (n = 74) [ASP needles at ear upper limb ear points] Usual care (n = 78)	Overall pain levels unchanged at any time point; modified BFA does not change pain, opioid use, or quality of life in those with lower-extremity surgery.
Liu et al. 2019 [71]	Nonpharmacologic interventions n = 182	Primarily pediatric and adolescent athletes: Acute sprains Elective surgery Appendectomy or extremity surgery	Acupuncture with e-stim vs no tx (n = 72) Hypnosis vs no hypnosis (n = 50) Imagery relaxation vs no intervention (n = 60) 15- to 30-minute txs	Acupuncture, hypnosis, and relaxation beneficial. Acupuncture with e-stim improved pain relief for athlete sprains.
Schiff et al. 2019 [72]	Nonpharmacologic n = 1127	Perioperative pain, nausea, anxiety	SOC+ acupuncture or reflexology or guided imagery (n = 916) SOC (n = 211) (do not give n for each intervention group)	SOC insufficient; acupuncture better than reflexology for nausea; otherwise, all therapies provided equal advantage to SOC for pain and anxiety.
Jan et al. 2020 [73]	BFA n = 90	ED acute abdominal, low back pain, or limb trauma.	SAC (n = 30) BFA+ SAC= Adj-BFA (n = 30) Sham+ SAC= Adj Sham (n = 30) Intervention provided by nurses, nurse practitioners, physicians, trainees	No significant differences across groups. BFA cannot be recommended for acute pain in ED. (BFA is an abbreviated form of acupuncture.)
Skonnord et al. 2020 [74]	Abbreviated, short, single tx of “Western medical” acupuncture protocol plus movement: n = 167 Acupuncture = 86	Acute nonspecific LBP; 11 primary care settings	n = 171 2 lumbar (right) hand points strong de qi; then patient mobilization movements 2 minutes, then 6 needles at Huatuojiaji L2–L4 segments to de qi (tx time 8–9 minutes) plus usual care vs SOC: advice regarding activity and medications (paracetamol and/or ibuprofen), and information on sick leave (Norwegian national guidelines).	No difference in pain relief across groups. UC time to recovery = 14 days. Acupuncture care plus UC time to recovery = 9 days. Though an abbreviated tx, meets 3-day threshold of clinical relevance, but authors inexplicably conclude it is not clinically relevant.

Adj = adjunct; AE= adverse event; EAS = electroacupuncture stimulation (e-stim on needles inserted at acupoints; e-stim  = electrical stimulation; nonpharm = nonpharmacologic; OR = operating room; pharm = pharmaceutical; SAC = standard analgesic care; SOC = standard of care; TENS = transcutaneous electrical nerve stimulation; tx = treatment; UC = usual care; VAS = visual analog scale; VNRS = verbal numerical rating scale.

**Table 6:** Acupuncture for acute LBP: SRs with/without meta-analysis

Authors, Year	Modality	SR	Meta-Analysis	Setting/Condition	Outcomes/Comparators	Results	Quality and Recommendation
Lee et al. 2013 [82]	Acupuncture 8/11 manual, EA, modern, wrist ankle	11 RCTs	7 RCTs	Outpatient, acute LBP, n = 1,139	3 acupuncture vs nonpenetrating sham 7 acupuncture vs NSAID medication 2/7 acupuncture, acupuncture+ meds vs meds alone 1 acupuncture + meds vs meds alone 6 “cured, improved, or failed” scale 6 NRS or VAS 4 function, 2 physical exam, 2 analgesic use	Acupuncture may be more effective than NSAIDs for global assessment, but effect is small. Acupuncture more effective than sham in reducing acute pain but not so for function or subacute pain. Acupuncture plus meds more effective for pain relief and overall function than meds alone. Fewer side effects than NSAIDs.	Quality mixed and needs consistency. Evidence shows potential for acupuncture, but further study needed to establish whether benefit compared with NSAIDs reflects evidence of equivalence. More research needed to establish optimal dose and frequency of acupuncture.
Chou et al. 2017 [81]	Nonpharm including acupuncture	11 RCTs of Lee et al. plus 2 RCTs	NA	n = 1,163 acute LBP (actually 1,139)	Acupuncture vs no acupuncture Acupuncture vs sham Acupuncture vs meds Acupuncture vs acupuncture plus meds vs meds Pain and function measures	Acupuncture decreased pain intensity more than sham, no clear impact on function. Greater likelihood of improvement compared to NSAIDs at end of tx.	SOE low to moderate for chronic LBP; SOE low for acute LBP. There is limited evidence that acupuncture is effective for acute LBP in short term (less than 3 months) and on a small to moderate magnitude. More evidence needed for acute LBP, to understand incremental benefits of combining and sequencing interventions.
Xiang et al. 2020 [95]	Acupuncture 4/14: 1 scalp acupuncture, 3 body acupuncture	14 RCTs n = 2,110 4 trials (sub) acute LBP (<12 weeks)	9 RCTs	4 acute LBP in outpatient setting (n = 753)	Acupuncture vs sham vs placebo vs UC Acupuncture vs sham	Moderate evidence of efficacy for acupuncture in terms of pain reduction immediately after tx for NSLBP ([sub]acute and chronic) when compared with sham or placebo acupuncture. Only minor AE.	Quality moderate. Need for research on specific techniques used, including needle placement, stimulation, needle depth, and the experience of the acupuncturists. Recommends standardization of the outcome measures and focus on duration/frequency of acupuncture sessions in future studies.
Su et al. 2021 [96]	Manual acupuncture, EA, AA	13 RCTs n = 899	13 RCTs n = 899	Settings not described. Acute LBP	Acupuncture (manual acupuncture, EA, ear acupuncture) vs drugs or sham acupuncture	Acupuncture significantly benefits VAS score (pain), ODI score, and NOP. Effect on RMDQ equal to controls.	Quality moderate. Acupuncture significantly benefits acute LBP symptoms, including reduction in analgesic medication. Heterogeneity of trials contributes to cautious recommendation of acupuncture for acute LBP. More research is needed.

EA = electroacupuncture; NA = not applicable; nonpharm = nonpharmacologic; NOP = number of pills; NRS = numerical rating scale; NSLBP = nonspecific low back pain; ODI = Owestry Disability Index; RMDQ = Rowland-Morris Disability Questionnaire; SOE = strength of evidence; tx= treatment; UC= usual care; VAS = visual analog scale.

**Table 7:** Acupuncture for acute migraine: SRs with and without meta-analyses

Authors, Year	Modality	SR	Meta-Analysis	Setting/Condition, n	Outcomes/Comparators	Results	Quality and Recommendations, Next Steps
Pu et al. 2016 [102] (Chinese language)	Acupuncture	NA	5 trials	Acute migraine n = 618	At 2 hours and 4 hours in acute migraine.	Acupuncture could effectively relieve the intensity of pain in acute migraine.	Quality unclear. Analgesic effect of acupuncture is significantly superior to sham acupuncture.
Coeytaux et al. 2016 [103]	Acupuncture	Overview of SRs	Overview of meta-analyses	Migraine, HA prevention: Cochrane SR (n = 22 studies, n = 4,985 participants) Tension-type HA: Cochrane SR (n = 12 trials, 2,349 participants) Chronic HA	HA frequency and response; compared with routine care (n = 5 studies); sham acupuncture control (n = 15); prophylactic drug tx (n = 5) HA response and number of HA days; compared to routine care (n = 2); sham acupuncture (n = 7); physiotherapy, massage or relaxation (n = 4) Specific outcome is unclear; compared with sham acupuncture (n= unclear)	Significant improvement in HA frequency compared with routine care and with prophylactic drug tx at 2 months. Acupuncture was significantly superior to routine care and sham acupuncture for response and reduction in HA days at 2, 3–4, and 5–6 months. Significantly larger effect size compared with sham acupuncture.	Quality not assessed. Acupuncture should be tx option to prevent migraine. Acupuncture should be a tx option for frequent episodic or chronic tension HA. None stated.
Zhang et al. 2019 [104]	Acupuncture	Overview of 15 SRs	Overview of 15 meta-analyses	Acute and preventive tx of migraine (n = 13 migraine; n = 1 included episodic migraine; n = 1 menstrual migraine included)	n = 15 VAS, clinical outcome, frequency Controls= no acupuncture, sham acupuncture, drug tx	n = 6 acupuncture superior to drugs; n = 4 acupuncture superior to sham acupuncture, drugs; n = 3 acupuncture superior to sham acupuncture; n = 1 acupuncture superior to drugs, other TCM txs; n = 1 acupuncture superior to tx migraine but did not mention control group in conclusions.	Methodological quality low. Acupuncture has advantage in pain improvement of VAS score, HA days/frequency, analgesic use, and efficacy of response rate. Poor quality of studies indicates better-quality research needed.
Li et al. 2020 [105] Overview of SRs	Acupuncture (body acupuncture, EA, ear acupuncture, warm acupuncture, scalp acupuncture)	Overview of 15 SRs	NA	n = 15 SRs	Sham acupuncture, placebo, medicine, other nonpharmacologic therapy, wait list. Primary outcome: effective rate. Secondary outcomes: intensity, frequency, duration of HA; use of painkiller, quality of life, recurrence, AEs.	AMSTAR 2 rating: 14/15 critically low-quality rating and 1 low quality. PRISMA-A: 11/15 adequately reporting over 70%. GRADE: high-quality evidence of acupuncture being superior to Western medicine (fewer HA days and painkiller uses, reduced frequency and HA degree compared with Western medicine or sham acupuncture).	High-quality evidence using GRADE tool. Acupuncture could be an effective and safe therapy for migraine, but quality of SRs need to be improved.
Yang et al. 2020 [106]	Acupuncture or acupoint stimulation with needle, heat, electricity, pressure, laser	13 trials n = 826	9 trials	Menstrual migraine	Sham devices; routine care; medications; acupuncture with medications. Primary outcome: number of migraines per month at completion of acupuncture tx. Secondary outcomes: days with migraine per month; mean HA intensity by VAS; medication use; frequency of migraines per month 3–6 months follow-up; AEs.	Acupuncture was not superior to sham acupuncture to reduce monthly migraine frequency and duration, intensity, or analgesic use. Pooled data: significant improvement in mean HA intensity in acupuncture group compared with drugs. Studies were underpowered, moderate to high risk of bias. No AEs.	Quality moderate. No strong evidence to support acupuncture in tx of menstrual migraine.
Natbony and Zhang 2020 [98]	4 ear acupuncture methods; 1 body acupuncture	Nonsystematic review	NA	Acute migraine (5) ED setting; episodic migraine prevention outpatient (1 SRM, 2 trials); chronic migraine prevention outpatient (3).	Pain reduction for acute migraine; reduction in migraine days in episodic and chronic migraine. Compared with various medications.	Acupuncture has potential for acute migraine in ED; acupuncture appears more effective than no tx or sham for prevention of episodic migraine. More study is needed for chronic migraines and to address barriers to access for acute migraines. Effective dosage and frequency of tx overall needs to be addressed in trials and the duration of benefit.	Quality not assessed. Acupuncture is a valid option for prevention of episodic migraines and has potential in ED for acute migraines.
Halker et al. 2020 [29]	Overview acute tx for episodic migraine (including acupuncture)	Included 4 acupuncture trials	NA	Outpatient, acute migraine, n = 475	3 trials compared with placebo; acupuncture superior to placebo on pain scale at 1 day.	Acupuncture could improve acute migraine pain compared with sham.	SOE low for acupuncture. More research is needed. SOE low or insufficient for opioids for acute migraine.

AE = adverse event; AMSTAR 2 = Measurement Tool to Assess Systematic Reviews; GRADE = Grading of Recommendations Assessment, Development, and Evaluation; HA = headache; NA = not applicable; PRISMA A = Preferred Reporting Item for Systematic Review and Meta-analysis-Acupuncture; SOE = strength of evidence; TCM = traditional Chinese medicine; tx = treatment.

### Acupuncture Therapy for Acute Pain

Acupuncture is understood as the insertion and manipulation of fine solid-core needles at specified points or combinations of points on the body. “Acupuncture therapy” derives from the traditional East Asian paradigm recognizing the interrelationship of organs, body points, and channels, as well as associated symptoms, dysfunction, and disease. Depending on the definition of the scope of practice in each state, acupuncture often includes treating by means of mechanical, thermal, or electrical stimulation; by the insertion of needles; or by the application of heat, pressure, or other forms of stimulation. In practice, acupuncture needling is deployed by first assessing a complex intersection of body organs, function, presentation, and interaction with a patient. It also is often done in combination with other therapies, such as palpation, Tui na, Gua sha, cupping, moxibustion, e-stim, auricular treatment, herbal medicine, and recommendations on diet, exercise, self-reflection, and meditative movement like Tai chi. Acupuncture therapy, therefore, includes an evaluation and decision-making process, acupuncture needling, accompanying therapies, and recommendations that engage a patient in self-care.

Significant research has focused on acupuncture in the treatment of pain. An SRM of immediate analgesic effects (13 randomized controlled trials [RCTs], n = 1,077) found that acupuncture was associated with greater immediate pain relief 30 minutes or less after the end of a single treatment than the pain relief reported with sham or analgesic injections [30]. A large individual patient data meta-analysis (39 trials) of 20,827 patients with chronic pain found acupuncture to be significantly better than sham treatment or usual care, with 85% retention of treatment benefit 1 year following a course of care [31, 32]. Patients with more severe pain at baseline improved more from acupuncture treatment than did those with lower levels of pain, compared with sham or non-acupuncture controls [33].

To be clear, the severity of pain is a predictor of response to acupuncture in chronic pain—i.e., the worse the pain, the better the response [33]. The immediate analgesia from a single acupuncture treatment provides better pain relief than does sham or analgesic injection, making acupuncture especially beneficial for acute pain, with opioid-sparing potential in hospital settings [30]. The largest hospital accreditation organization in the United States, The Joint Commission, has revised its pain mandate, which was originally introduced in 2000. Effective January 1, 2018, The Joint Commission requires its accredited hospitals and facilities to provide nonpharmacologic strategies for pain as a scorable Element of Performance [10]. In support of these efforts, the present narrative review includes SRs and SRMs of acupuncture for inpatient postoperative pain, as well as for acute pain not related to surgery, acute pain in an ED setting, and acute pain in primary care [6]. We include the details of the effect sizes and quality of reviews as reported by the authors, but we do not systematize authors’ reporting of results.

#### Acupuncture Therapy for Postoperative Pain

In multiple SRMs, acupuncture was found to be effective in reducing postsurgical pain compared with sham acupuncture, controls, and usual care, with a reduction in opioid need (21% opioid reduction at 8 hours, 23% at 24 hours, and 29% at 72 hours post surgery) and with a lowered incidence of opioid-related side effects such as nausea, dizziness, sedation, pruritus, and urinary retention [34–36]. An SRM found that acupuncture after total knee arthroplasty reduced pain and was associated with delayed opioid use [37]. An SRM of perioperative auricular acupuncture found reduced postoperative pain and need for analgesic use compared with sham or standard-of-care controls [38]. Another SRM on perioperative auricular therapies found benefit for pain and intraoperative body mass–adjusted fentanyl amount but did not find prolonged time to first analgesic request in total hip arthroplasty [39]. An SRM of auricular acupuncture for acute and postsurgical pain found that it provided immediate pain relief and benefit at 48 hours, was equivalent to analgesics, and had fewer side effects [40]. These findings indicate the potential to reduce hospital readmission due to uncontrolled pain. (See Table 1.)

A 2015 SRM of acupuncture point stimulation for postoperative pain (59 RCTs; n = 4,578) showed significantly improved visual analog scale (VAS) pain scores, with a further benefit of reduced total morphine consumption [34]. Still, a 2016 clinical practice guideline on postoperative pain management by the American Pain Society, the American Society of Regional Anesthesia and Pain Medicine, and the American Society of Anesthesiologists’ Committee on Regional Anesthesia did neither “recommend nor discourage” acupuncture therapy as part of recommended multimodal postoperative pain care. The guideline recommendation was based on only six studies that used active sham arms [41], which are not inert controls and therefore constitute a flawed methodology [42, 43]. A subsequent SRM (2016) supported the use of acupuncture as adjuvant therapy in treating postoperative pain and for reducing opioid use [35]. A 2019 SR found that distal acupuncture needling compared favorably with peri-incisional transcutaneous electrical nerve stimulation in pain reduction and opioid sparing for patients having open abdominal surgery [44].

Trials published since 2016 include a double-blind placebo-controlled trial in which intraoperative electrical stimulation of acupuncture points reduced intraoperative opioid requirements, postoperative pain, and duration of stay in the post-anesthesia care unit [45]. Another double-blind placebo-controlled trial showed that the addition of transcutaneous electrical acupoint stimulation (application of electrical stimulation at acupuncture points) to usual care for minimally invasive lung cancer surgery reduced pain, reduced patient-controlled intravenous analgesic attempts, and reduced nausea and vomiting, which supports transcutaneous electrical acupoint stimulation as a feasible option for sedation and postoperative analgesia in thoracoscopic pulmonary resection [46].

Additionally, acupuncture was effective, safe, and well tolerated for post-tonsillectomy pain in children, with no significant side effects [47].

#### Acupuncture Therapy for Acute Pain in the ED/ICU 

As of 2021, for acute pain in the ED or urgent care setting, there were six SRs of acupuncture [50–53], of which three had meta-analyses (Table 2) [51, 53, 54]. Of these, one was for ear acupuncture alone [54]. Reviews included trials of acupuncture in the treatment of various acute pain conditions and injuries in adults and children in the ED, including acute LBP, acute neck pain, and acute ankle sprain; other musculoskeletal pain conditions, fractures, and nonpenetrating injuries; acute abdominal pain, appendicitis pain, renal colic, acute headache and migraine, acute dental pain, and acute pharyngitis.

One SRM included 19 RCTs and 11 observational studies and reported that acupuncture provided effective analgesia for most acute pain conditions in the ED while being noninferior to selected analgesia medications and providing statistically significant, clinically meaningful, and improved levels of patient satisfaction with respect to pain relief in the emergency setting [51]. The authors concluded that acupuncture was low cost and low risk with high patient satisfaction. In addition, there was one review of nonpharmacologic approaches to pain in the ICU, including acupuncture (see Table 3) [55].

Among studies not in SRs or SRMs (Table 4), a trial of patients in the ED found acupuncture’s benefit to be comparable to pharmacotherapy for acute LBP (n = 270) or ankle sprain (n = 166) and found that acupuncture was safe [56]. In an observational study of 1,008 patients including children in a surgery ward, acupuncture given as first aid immediately after or optimally within 48 hours of a burn injury reduced pain, reddening, pigmentation, scarring, and post-traumatic stress disorder, which commonly follows traumatic burns [57]. Two randomized trials found that acupuncture for acute pain in the ED performed better than titrated or intravenous morphine, with minimal adverse effects [58, 59].

Studies also reported high acceptability for acupuncture by patients with acute pain in the ED, when measured [56, 60, 61]. A retrospective study of patients with acute pain in the ED found that acupuncture produced a decrease in pain comparable to that produced by analgesics, with the additional benefits of reduction in anxiety as well as high acceptability among both medical providers and patients (Table 5) [62]. Seventy-five percent of providers referred for acupuncture, and 90% of patients agreed to acupuncture. When details were reported in studies, acupuncture sessions for acute pain in the ED averaged 10–30 minutes [58, 63], with mean session durations of 23–24 minutes [60, 62], and they did not disrupt ED course of care [60–62].

**Table 5:** Pilot, retrospective, or qualitative studies: acupuncture for inpatient or ED acute pain

Author/year	Modality/kind of study/n	Setting/type of pain	Intervention/comparator/Outcome measures	Results
Crespin et al. 2015 [75]	Acupuncture/retrospective OBS/n = 2,500	Postoperative pain care after total hip or total knee replacement	Elective acupuncture as adjunct to physical therapy beginning first day after surgery. All Patient Refined-Diagnostic Related Groups (APR-DRG) severity of illness measures; self-reported pre- and post-tx pain scores 0–10	Nearly 75% of patients elected to have acupuncture in addition to PT; acupuncture reduced pain by 45% in short term and improved patients’ capacity to perform PT during initial postsurgical recovery.
Quinlan-Woodward et al. 2016 [76]	Acupuncture/pilot RCT/n = 30	Inpatient / post–breast cancer surgery	Acupuncture (n = 15) UC (n = 15) NRS for pain, nausea	Pain, nausea, and anxiety were reduced in acupuncture group on the first day, and pain was also reduced on the second day after surgery.
Reinstein et al. 2017 [62]	Acupuncture Retrospective OBS n = 248	Pain and anxiety ED Back (n = 57) Head (n = 41) Limb (n = 37) Abdomen (n = 27) Chest (n = 17) Groin (n = 3)	Feasibility outcomes: 248/279 = 89% of patients agreed to acupuncture. 55/75 = 73% of clinical providers referred patients for acupuncture. Acupuncture sessions averaged 23 minutes (SD 8.9) and ranged from 6 to 78 minutes. Acupuncture tx vs UC analgesics. NRS 0–10 for pain and anxiety.	Acupuncture acceptable and effective for pain and anxiety reduction with standard care. Of patients with pre pain (n = 182), 43% reported ≥50% pain reduction, and 57% reported ≥30% pain reduction. Similar benefits were seen regardless of whether any pain medication also was received in the ED (n = 88) vs acupuncture alone (n = 92).
Burns et al. 2019 [60]	Acupuncture Retrospective OBS n = 379	ED acute pain: Neck/back/shoulder/hip (n = 133) Abdominal pain including urinary tract and gastric (n = 123) Chest pain, including anxiety/hypertension related (n = 35) Head pain (including headache, Bell’s palsy, epistaxis, trigeminal neuralgia) (n = 37) Joint/limb pain (n = 31) Substance withdrawal pain (n = 6) Generalized pain (all over or more than one site) (n = 14)	Acupuncture: 53.7% of 706 patients agreed to acupuncture (n = 379). 86% had 8–15 needles. 92.6% had 20- to 30-minute needle time (mean 24.4 minutes) Pre- and post-acupuncture pain, stress, anxiety, and nausea scores.	Acupuncture is feasible and acceptable for acute pain patients in ED. Patient-reported pain, stress, and anxiety scores all significantly improved after acupuncture, with similar benefits seen regardless of whether any pain medication also was received. Receiving only opioids during ED visit was not associated with improved pain scores. AEs not reported.
Aikawa et al. 2020 [77]	Acupuncture OBS n = 102	ED, acute musculoskeletal pain (n = 102) LBP, neck pain, knee pain, shoulder pain.	10-second intense tx at single or 2 acupoints NVS before and after tx. SI 3, BL 62, GB 41.	Almost all reported decrease in pain; only 4% had desire for analgesic medication.
Tsai et al. 2020 [78]	Acupuncture Retrospective study n = 24	Outpatient and inpatient units; pediatric sickle cell pain	90 txs/24 patients, mean tx duration 18.5 ± 4.8 minutes Pre/post pain scores	No AEs. Pain reduction.
Mahmood et al 2020 [79]	Acupuncture retrospective n = 12	Inpatient and outpatient units, pediatric sickle cell pain	Adjuvant acupuncture 15–20 minutes	Acceptable, feasible; improved pain.
Tsai et al. 2020 [80]	Acupuncture manual (75%) Electroacupuncture (1%) Combined manual and electro (24%)	Outpatient, migraine	Acupuncture (n = 477), mean 8.9 sessions, with medications Medications alone (n = 1,908): sumatriptan, rizatriptan, ergotamine, caffeine, acetaminophen, ibuprofen, and other NSAIDs	In migraine patients who underwent acupuncture tx, the medical expenditures on emergency care (*P*=0.01) and hospitalization (*P*=0.01) were significantly lower than for patients without acupuncture tx. It is cost-effective to encourage combining acupuncture and Western medicine to treat migraine patients.

AE = adverse events; NRS = numerical rating scale; NSAIDs = non-steroidal anti-inflammatory drugs; NVS = numerical visual scale; OBS = observational study; PT = physical therapy; SD= standard deviation; tx = treatment; txs = treatments; UC= usual care.

Acupuncture for acute pain in the ED is a developing area of research focus, based on single treatments during a narrow window of time. The details in Table 2 include outcome measures in addition to comparators.

#### Acupuncture for Acute LBP 

There is evidence that acupuncture is effective for acute LBP in the short term (for 3 months or less) and on a small to moderate magnitude [81]. The ACP recommends acupuncture as a first-line treatment for acute and subacute LBP on the basis of their 2017 SRM (11 RCTs, n = 1,163; actually n = 1,139) [13, 82]. That review was based primarily on a 2013 SRM by Lee et al. (11 RCTs, n = 1,139) [82] that found acupuncture to be more effective than sham for acute LBP, but not for function, and comparable to nonsteroidal anti-inflammatory drugs (NSAIDs) for pain with reduced risk of side effects. The authors (2013) called for noninferiority research to clarify equivalency with NSAIDs. The ACP SRM considered two RCTs for acute LBP in addition to the 2013 SRM by Lee et al.: Hasegawa et al. (2014) (n = 80) [83] and Vas et al. (2012) (n = 275) [84]. Hasegawa et al. compared a Japanese scalp needling technique with traditional Chinese body acupuncture. The body acupuncture was not detailed but was not an “inert” intervention and so not a true sham control. The trial used one acupuncturist, a specialist in the scalp technique. Although the scalp technique showed benefit relative to body acupuncture in some measures, the results are questionable on the basis of the problematic design [83]. Vas et al. reported real and sham acupuncture to be superior to placebo at 3 months but equal to sham and placebo compared with medication alone at 12 months [85]. However, the study was powered only to detect differences between true acupuncture and the medication control group, where true acupuncture was superior to medication control. It was not powered relative to sham or placebo. Moreover, points used in the sham group are reported in the literature to be effective for LBP, making the sham arm an active acupuncture treatment [86]. Inclusion of Hasegawa et al. and Vas et al. in the ACP review is problematic and potentially misleading given these sham control design limitations.

The benefit of acupuncture for acute LBP is also informed by trials in the ED or urgent care setting. A multicenter equivalence, noninferiority RCT (n = 528) of acupuncture for acute LBP in the ED found acupuncture to be safe, acceptable, and comparable to medications (n = 270) (Table 4) [56]. A 2020 study (n = 167) of a single abbreviated acupuncture treatment (8–9 minutes) for acute LBP that used hand points, movement, and several back points showed that it provided no advantage in pain relief compared with medications but did shorten the time to recovery from 14 to 9 days [74]. Although this recovery time benefit reached the authors’ threshold, inexplicably, they reported it as not clinically significant.

The abbreviated auricular treatment known as battlefield acupuncture (BFA) did not provide additional benefit for acute pain, including acute LBP, in the ED [73]. There may be an advantage to more comprehensive acupuncture treatment that is responsive to individual patient presentations [56] over rote abbreviated techniques like BFA [73, 74]. Moreover, BFA has been marketed as a “battlefield” option for acute pain, but it has not been studied in that setting. It is used primarily for chronic pain within the Veterans Health Administration outpatient care. Although BFA has benefited from auriculotherapy research [87], it has not been studied relative to other auricular protocols, nor is there any proven advantage of ASP^®^ needles (ASP: Aiguille SemiPermanent needles Sedatelec, Chemin des Mûriers 69540 Irigny, France) over other intradermal needles or ear seeds for extended auricular therapy, as has been claimed by BFA Seminars [88, 89]. Rather, the “harpoon”-shaped needles [90], which are designed to embed in the chondral layer of the ear, could increase the risk of harms [91]. Auricular acupressure with *Vaccaria* seeds fixed to ear points has been shown to be as effective as, if not more effective than, any retained intradermal needles, with a greatly reduced risk of harms [87].

Trials of acupuncture for specific kinds of acute back pain also illuminate its potential. An SRM on acute pain in the ED that included three trials of acupuncture for renal colic found acupuncture to be as effective as medication with fewer side effects (n = 38) [92], making it a potent option for immediate renal colic pain while avoiding the risks of NSAIDs or acetaminophen (n = 121) [93]. Acupuncture was as effective as Dolantin (meperidine) but superior to Scopolamine (hyoscine) in another trial on renal colic (n = 240), with onset time to pain relief significantly earlier for acupuncture and with greatly reduced adverse reactions [94]. Since the 2017 SRM, another trial (n = 115) found acupuncture to be superior to titrated morphine for renal colic in the ED, with significantly sooner onset of pain relief (50% reduction), deeper pain relief, and greatly reduced adverse effects [58].

#### Acupuncture Therapy for Acute Headache and Migraine

Acupuncture has been studied for acute migraine [97], migraine prophylaxis and prevention, and impact on medical expenditure [80]. Table 7 details the SRs and SRMs that evaluated acupuncture for acute headache/migraine. Although existing research indicates that acupuncture could be a potential strategy for acute migraine, where its benefit exceeds that of sham acupuncture [29, 98], comparing acupuncture with protocol medications in the ED setting presents unique challenges. Acupuncture is not readily available in ED settings in the United States to conduct research, but more importantly, standard medical protocols preclude engaging acupuncture as a strategy for acute migraine in the ED [99]. For example, opioids are one standard option in the ED that precludes an acupuncture intervention, even though the evidence base for opioids’ effectiveness and safety for acute migraine remains limited [29].

Where migraine and headache carry a significant global economic burden [100], real-world data from a 10-year national cohort study in Taiwan showed that in patients with migraine who received acupuncture treatment, medical expenditures on emergency care and hospitalization were significantly lower than those of the group without acupuncture treatment [80]. A review of 10 years of RCTs of acupuncture vs various controls in the treatment of migraine (49 trials) found that acupuncture reduces symptoms of migraine and improves quality of life [101]. The meta-analysis showed that acupuncture reduces headache frequency more than no treatment or medications, but the quality of evidence was low. For acute migraine (three trials), real acupuncture had a greater effect on reducing headache-related pain as measured by visual analog scale (VAS) score, earlier onset and longer duration of headache relief, and greater clinical effectiveness immediately after treatment compared with the sham acupuncture control. To reduce migraine symptoms and frequency as well as medical expenditures for migraine-related emergency care and hospitalizations, authors recommend combining acupuncture with usual care [80]. 

## Discussion

The need for evidence-based comprehensive pain care strategies is based on insufficient management of acute pain, continued reliance on opioids for acute pain, the risk associated with opioids, and the ongoing rise in opioid deaths. This review of reviews and meta-analyses supports acupuncture therapy as a viable strategy for acute pain care.

### Acute Perioperative, ICU, and ED Pain

Reviews of acupuncture for acute perioperative pain related to various surgeries, including major surgeries, are listed in Table 1. Eleven SRs, 10 of which have meta-analyses, detail the feasibility and benefits of acupuncture. Nine reviews also tracked a reduced need for analgesic medication, including opioids. This is a substantial finding, given the risk of even short-term opioid use for acute pain [18].

For acute pain in the ED, six SRs, three of which have meta-analyses, demonstrate increasing interest in and feasibility and acceptability of acupuncture in the ED (Table 2) and ICU (Table 3). Table 5 details pilot, observational, retrospective, or qualitative studies on acupuncture’s reduction of acute pain, high level of acceptance, and low risk of adverse events [60, 62, 75–80].

Additional trials that evaluated acupuncture for acute pain in the ICU for surgical patients, inpatients, or patients in the ED are detailed in Table 4. Two studies evaluated acupuncture for pain in intubated ICU patients: one using electrical stimulation on needles inserted into acupuncture points [65] and the other using transcutaneous electrical nerve stimulation on acupuncture points [68]. The former found a marked reduction in the dosage of the sedative drug (midazolam) needed for the pain and discomfort of mechanical intubation [65]. The latter found a reduction in pain and in analgesic and sedation medication [68].

Three studies evaluated a trademarked, abbreviated version of auricular therapy, called BFA, for acute pain, with mixed results. A small study found the addition of BFA for acute LBP to be feasible and effective [69], on par with auricular therapy in general [87]. In other studies, BFA for acute pain in lower-extremity surgery (n = 233) and for acute abdominal, low back, or limb pain in the ED (n = 90) was found to be ineffective [70, 73]. Another trial using a different abbreviated acupuncture protocol for acute LBP also found no benefit [74]. However, a larger multicenter trial using a more robust acupuncture protocol for acute pain in the ED (n = 528) found that acupuncture provided analgesia comparable to that provided by medication for acute LBP and ankle sprain [56].

Two additional studies evaluated acupuncture as a nonpharmacologic pain strategy in the acute or perioperative setting. Acupuncture for pediatric/adolescent patients (n = 182) reduced pain [71]. The other study of perioperative adults (n = 1,127) treated with acupuncture resulted in reduced pain, nausea, and anxiety [72].

### Acute LBP and Migraine

Four reviews of acupuncture for acute LBP, three of which had meta-analyses, are detailed in Table 6. The most recent review and meta-analysis (2021) [96] found that acupuncture significantly benefits acute LBP symptoms, including a reduction in analgesic medication.

Finally, Table 7 details seven acupuncture reviews for acute migraine: two with meta-analyses and two with overviews of meta-analyses. Acupuncture had a significantly larger effect than sham [29, 102, 103] and a larger effect than medications [103–105]. Acupuncture appears to be more effective than no treatment or sham in the prevention of acute migraine, with potential as a valid option in the ED [98]. Although a review of acupuncture for menstrual migraine found a reduction in mean headache intensity from pooled data, it did not find acupuncture to reduce monthly migraine frequency, duration, or analgesic use [106].

### Limitations

This narrative review allows for the inclusion of diverse articles with substantial findings on acupuncture for acute pain across various clinical settings, allowing clinicians and policy makers to review the breadth and depth of the literature. Some of the cited SRMs include trials that compared acupuncture with active sham interventions, a problematic methodology wherein controls that are not “inert” can produce physiological changes [31, 42, 43, 107, 108]. When reporting on these studies, we indicate the authors’ quality assessments, but in some cases, they are at risk of underestimating the true value of acupuncture because of the active nature of the sham acupuncture used [42]. Details of effect sizes and the quality of reviews are included as reported by the studies’ authors, but it is beyond the scope of the present narrative review to systemize authors’ reporting of results.

The potential for acupuncture to avoid or reduce opioid use in acute pain is reported in some reviews, and these data should not be undervalued. As few as 5 days of prescribed opioids as the initial treatment of pain increases the probability of long-term opioid use [18]. Even the Centers for Disease Control and Prevention recommends non-opioid and nonpharmacologic options as a first line of treatment for acute postsurgical pain [16]. Against the background of the ongoing crisis of opioids, addiction, and death due to opioids, metrics on analgesic use, including opioids and NSAIDs, should be encouraged as a clinically meaningful outcome measure in future studies. Future multisite trials to clarify the scope, timing, and generalizability of acupuncture for acute pain the ED are recommended. On the basis of research that previously established the advantage of multidisciplinary pain care, future research must focus on individual as well as combination strategies for comprehensive pain care, which include evidence-based nonpharmacologic strategies in the acute pain care setting.

### Acupuncture Therapy Safety

Acupuncture has a low risk of adverse events. More specifically, the associated effects of acupuncture have been categorized as secondary benefits, such as feeling relaxed, elated, or tired or having improved mood and sleep. The National Institutes of Health Consensus Statement on Acupuncture published in 1998 found that “… the incidence of adverse effects is substantially lower than that of many drugs or other accepted procedures for the same conditions” [109]. SRs and surveys have clarified that acupuncture is safe when performed by appropriately trained practitioners [110–117], with infrequent minor side effects, such as feeling relaxed, elated, or tired or having sensation or itching at the point of insertion [114]. Rare serious complications, such as infection or pneumothorax, are directly related to insufficient training [115, 116, 118]. Safe use of acupuncture has also been established in vulnerable populations, including children [110, 119–121] and pregnant women [122–124]. Active military service members who accessed acupuncture for chronic pain had a reduced risk of long-term adverse outcomes [125].

Research needs to continue to carefully track and report any adverse events associated with acupuncture and compare them with adverse events associated with medications used, such as opioids. Acupuncture’s reduced risk is apparent in general, but one article serves as a relevant example. In the management of acute pain in the ED, acupuncture (n = 150) was compared with titrated morphine (n = 150) [59]. Of the 300 participants, adverse effects were experienced by 89 (29.3%) patients: 85 (56.6%) in the morphine group and 4 (2.6%) in the acupuncture group. Primary adverse events in the morphine group were dizziness, nausea, and vomiting. Acupuncture had a better success rate, with faster resolution to 50% reduction of pain than was seen in the morphine group.

### Frequency, Dosage, and Timing of Nonpharmacologic Interventions for Inpatient and Acute Pain Care

Inpatient acupuncture therapy is usually delivered by a licensed independent practitioner, such as an acupuncturist, and is generally given as a daily treatment for the term of the inpatient stay, with referral for outpatient care follow-up. ED settings allow for a single treatment only. A session of inpatient or ED acupuncture care can last 20–60 minutes, with 30–45 minutes being typical. A session would likely include an interview assessment, palpation, point location, needling that may or may not include “de qi” response, resting with needles in place, needle removal and disposal, reevaluation, and recommendations. Needle retention times vary and could be 15–25 minutes depending on whether one aspect of the body or more is treated. If the front and back of the body are treated, needle retention might be 10–20 minutes for each side. If only one aspect is treated, needle retention might be longer. Body acupuncture typically involves points both local to and distal from the problem area.

Abbreviated treatments result in less optimal outcomes. For example, the limited auricular protocol BFA was not effective for acute abdominal pain, acute LBP, or limb trauma pain in the ED [73]. Modified BFA was also not effective as an adjunct for pain relief in lower-extremity surgery [70]. Another ED trial used an abbreviated 10-minute acupuncture treatment for acute LBP with usual care vs usual care alone, finding no difference in pain relief [74].

There are evidence-based data on the dosage and frequency of acupuncture for acute pain, and more research will be beneficial to further define the optimal frequency, dosage, and timing of care: What constitutes an optimal intervention in terms of session time, number of points palpated and needled, and needle retention time, and with what additional hands-on therapies? Parameters of effective acupuncture treatment for chronic pain could inform acute pain care. For example, in a large individual patient data meta-analysis (39 trials, n = 20,827) 95% of trials included local and distal points, 94% aimed to obtain “de qi,” 69% used 5–14 acupoints in a session (21% used 15–20 acupoints), 50% had mean session durations of 20–29 minutes, and 47% had mean session durations of 30 minutes or more [31]. The only patient characteristic that predicted outcome was severity of pain at baseline, wherein patients reporting more severe pain at baseline experienced more benefit from acupuncture compared with either sham control or non-acupuncture control [33]. These findings from such a large study may be generalizable to acute pain care.

### Multimodal Approach to Acute Pain Care

The era of the promise and promotion of opioids coincided with a retraction of insurance coverage for multidisciplinary pain care strategies, which in turn created a barrier to access to and funded research of nonpharmacologic therapies [126, 127]. Multimodal pain care is now recognized as the optimal, inclusive, and responsive approach to patients experiencing pain: inclusive of all evidence-based therapies, including effective nonpharmacologic strategies, and responsive to patients’ diverse and evolving needs [127]. Evidence-based nonpharmacologic therapies are recommended in comprehensive pediatric and adult pain care [10, 128, 129]. Multimodal pain care is recommended by the American Pain Society in their guidelines for postoperative pain management [41]. Effective nonpharmacologic strategies are recommended by the ACP in their guidelines for acute LBP [13]. Comprehensive multimodal pain care must include effective nonpharmacologic strategies such as acupuncture in the inpatient and acute care setting, with a seamless referral system to support ongoing care once a patient is discharged. Nevertheless, as clearly stated in the review of back pain by Cherkin et al, “… Despite improved knowledge about the benefits and harms of treatments for chronic back pain in the past several decades, there is a large and consequential mismatch between treatments found safe and effective and those routinely covered by health insurance … The barriers to change identified in the IOM (Institute of Medicine) and *Lancet* reports make it clear that deficiencies in our health care delivery and payment models are centrally involved in the continued failure to improve care for back pain” [130]. This deficit applies equally to all types of pain. There is a pervasive failure to align insurance coverage to ensure access to evidence-based, safe, and effective pain treatments [131].

### Acupuncture Biomechanisms

Scientific discoveries over the past several decades have demonstrated complex and robust biological mechanisms for the therapeutic effects of acupuncture. A full review of the biomechanism literature is not within the scope of the present article, and we aim only to highlight some of the most significant contributions of recent science. Research on acupuncture’s impact on the musculoskeletal system includes evidence that acupuncture needle insertion stimulation influences connective tissue and fascia, collagen production, and fibroblast alignment and activity [132–134]. Acupuncture has also been shown to reduce inflammation locally, which in turn impacts pain processing by the central nervous system [135–137]. There is robust research showing how acupuncture stimulates neuroplastic changes that are known to interfere with the processes of central sensitization that are well recognized to perpetuate chronic pain. The impact on the central and peripheral nervous systems includes neurochemical alterations of the endogenous opioid [138–140] and endocannabinoid systems [141, 142].

Neuroimmune mechanisms include alterations in the nuclear factor kappa B pathway and in the TRPV1 and TRPV2 channels of mast cells, as well as reduced levels of substance P (SP), neurokinin-1 receptor, interleukin-6, interleukin-1β, and tumor necrosis factor-α [143], thereby inhibiting the release of excitatory neurotransmitters while promoting the release of inhibitory neurotransmitters from neurons and glial cells. Acupuncture also regulates neuroinflammatory substances that inhibit microglial crosstalk [144, 145].

Central mechanisms of acupuncture include deactivation of the limbic system, which is important for emotion and internal homeostasis, which impact chronic pain [146–148]. Functional magnetic resonance imaging evidence demonstrates beneficial modulation of the maladaptive alterations in somatosensory cortical maps that have been shown to accompany chronic pain states and interfere with optimal motor recruitment patterns [149, 150].

As pain and physiology research have evolved, discoveries have demonstrated the interconnected roles of the immune system, the central and peripheral nervous systems, and all organ systems in the phenomena of nociception and its interpretation by the brain. This level of interconnectedness requires comprehensive strategies to understand pain, assess it, and deliver care. As iterated by John Bonica since the 1940s, pain care requires attention to mind and body and is best delivered through comprehensive strategies that were the cornerstone of his vision for pain treatment [151]. Evidence-based therapies such as acupuncture therapy have a vital role to play in comprehensive pain care.

## Summary

Our narrative review includes 22 SRs, of which 17 have meta-analyses, and it supports the feasibility and benefit of acupuncture therapy as an effective standalone or adjunct intervention in acute perioperative pain and acute pain in the ED or urgent care setting. The evidence supporting effectiveness, safety, reduced need for opioids and NSAIDs, and improved patient satisfaction is a compelling reason for acupuncture therapy to be covered for acute pain by public and private insurance. Policy barriers preventing licensed acupuncturists from being Medicare-billing providers need to be addressed. Health care practitioners and administrators need training in the evidence base for acupuncture therapy, and they need to advocate for policy initiatives that remedy systemic reimbursement barriers to evidence-based comprehensive pain care strategies. The support, promotion, and dissemination of ongoing research into the expanding role of effective nonpharmacologic treatments for pain will need to continue to address both the short- and long-term therapeutic and economic impact of comprehensive pain care practices. Multimodal pain care is now recognized as the optimal, inclusive, and responsive approach to patients experiencing pain: incorporating all evidence-based therapies, including effective nonpharmacologic strategies is responsive to patients’ diverse and evolving needs.

## Authors’ Contributions

All authors participated in searches for and evaluation of reviews and studies. All authors drafted the manuscript; AN and HT responsible for coordination of the finalized manuscript.

## Notes

Funding sources: This overview was supported by the Academic Consortium for Integrative Medicine & Health, Pain Task Force Project. Dr. Dusek’s work on this publication was partially supported by the National Center of Complementary and Integrative Health (NCCIH) of the National Institutes of Health under award number R01AT010598. The study sponsor had no role in the study design; collection, management, analysis, and interpretation of data; writing of the report; or the decision to submit the report for publication, including whether the sponsor will have ultimate authority over any of these activities. The content is solely the responsibility of the authors and does not necessarily represent the official views of the National Institutes of Health.
